# An Activity Recognition Framework Deploying the Random Forest Classifier and A Single Optical Heart Rate Monitoring and Triaxial Accelerometer Wrist-Band ^†^

**DOI:** 10.3390/s18020613

**Published:** 2018-02-22

**Authors:** Saeed Mehrang, Julia Pietilä, Ilkka Korhonen

**Affiliations:** 1 BioMediTech Institute and Faculty of Biomedical Sciences and Engineering, Tampere University of Technology, 33720 Tampere, Finland; julia.pietila@tut.fi (J.P.); ilkka.korhonen@tut.fi (I.K.); 2 Department of Future Technologies, University of Turku, 20500 Turku, Finland

**Keywords:** accelerometer, activity recognition, context awareness, machine learning, photoplethysmography, random forest, wrist-worn sensors

## Abstract

Wrist-worn sensors have better compliance for activity monitoring compared to hip, waist, ankle or chest positions. However, wrist-worn activity monitoring is challenging due to the wide degree of freedom for the hand movements, as well as similarity of hand movements in different activities such as varying intensities of cycling. To strengthen the ability of wrist-worn sensors in detecting human activities more accurately, motion signals can be complemented by physiological signals such as optical heart rate (HR) based on photoplethysmography. In this paper, an activity monitoring framework using an optical HR sensor and a triaxial wrist-worn accelerometer is presented. We investigated a range of daily life activities including sitting, standing, household activities and stationary cycling with two intensities. A random forest (RF) classifier was exploited to detect these activities based on the wrist motions and optical HR. The highest overall accuracy of 89.6 ± 3.9% was achieved with a forest of a size of 64 trees and 13-s signal segments with 90% overlap. Removing the HR-derived features decreased the classification accuracy of high-intensity cycling by almost 7%, but did not affect the classification accuracies of other activities. A feature reduction utilizing the feature importance scores of RF was also carried out and resulted in a shrunken feature set of only 21 features. The overall accuracy of the classification utilizing the shrunken feature set was 89.4 ± 4.2%, which is almost equivalent to the above-mentioned peak overall accuracy.

## 1. Introduction

Activity monitoring and detection is essential, e.g., in designing context-aware environments and physical therapies. Quantification of the intensity and type of the physical activities helps with understanding the individual’s lifestyle and facilitating the process of behavior change [1]. Lack of sufficient physical activity is directly related to higher risk of stress, cardiovascular disorders, diabetes and musculoskeletal disorders [2]. In the case of elderly adults, activity monitoring can play a key role in detection of long inactive time periods or fall events [3]. Inability to recover from a fall can lead to debilitation and even fatal outcomes [4]. Therefore, real-time activity recognition systems that are sensitive enough in detecting such critical time points are required [5].

For many years, wearable inertial measurement units (IMUs) have been used to detect human activities. However, in many studies, researchers have tested multi-sensor configuration or less compliant measurement sites such as hip, ankle, chest and waist, which are not practical for long-term use [6]. Wrist-based activity recognition using currently available smartwatches and wristbands seems more feasible and user friendly for long-term applications [3]. Detecting physical activities by means of wrist-worn devices is, however, more challenging due to the wide degree of freedom of hand gesticulations [6]. That is, the movement pattern of the hand varies widely and may look similar while different types of activities are carried out. For example, drinking while sitting reflects a similar hand movement pattern as drinking while standing. Such similarities in hand movement patterns hinder accurate activity recognition. This problem can be alleviated by providing additional information about the context. One of the available options is equipping the wrist-worn IMUs with heart rate (HR) sensors. HR can be measured using wrist-worn photoplethysmography (PPG) sensors. If triaxial accelerometers are also embedded, the device can be probably used for activity recognition purposes with better accuracies compared to conventional wrist-worn triaxial accelerometers. Hereafter, the PPG-ACC wristband refers to such wrist-worn devices that can acquire both PPG-based HR and acceleration (ACC) signals.

In this paper, we are aiming to present a comprehensive assessment of a human activity recognition (HAR) framework that will be suitable for both real-time and non-real-time applications. The framework covers some of the principal building blocks required for an activity monitoring system that works only based on a single PPG-ACC wristband. The system operates based on a well-known supervised classification algorithm, random forest (RF) [7], which has been widely tested in the literature [2,8,9]. The data processing comprised of label correction, signal segmentation, feature extraction, parameter examinations and feature reduction is comprehensively explained, and the corresponding strengths and weaknesses are discussed. We report the effect of window length, number of trees in the RF, effect of HR features, the optimal number of features and the most important HR and ACC features. Confusion matrices and the percentage of weighted mean accuracies (weighted standard deviations) are the criteria for benchmarking the goodness of the results.

To obtain the best parameter choices, a dataset of activities performed by 20 subjects was used. Two levels of cycling, different types of sitting, a series of household activities and standing still were successfully recognized and classified. For each parameter, a set of values was inputted to the RF, and the results were observed by leave-one-subject-out cross-validation (LOOCV). Having such a kind of comprehensive examination, the future implementations of the RF algorithm for wrist-based activity recognition become easier and more efficient. To the best of our knowledge, this is the first time that such a comprehensive activity recognition framework based on a single PPG-ACC wrist-worn sensor has been presented. Having both HR and ACC data simultaneously helps with recognizing varying intensities of the same activity, as will be shown in the upcoming sections.

The rest of the paper is structured as follows. Section 2 is comprised of the literature review and related work. Materials and methods are presented in Section 3. Experimental results are described in Section 4. In Section 5, we discuss the results. Finally, in Section 6, we present the conclusions.

## 2. Related Work

Wrist-based activity recognition became popular since the wrist-worn IMUs with enough battery and memory were designed. For instance, the survey of National Health and Nutrition Examination (NHANES) changed the location of accelerometer measurements from the hip to the wrist in 2011 [10]. One of the most prominent advantages of using wrist-worn sensors for activity recognition is better compliance compared to other measurement locations [9].

GENEActiv, a wrist-worn triaxial accelerometer, is one of the most popular wrist-worn activity tracking sensors and has been used in a number of relevant studies. In the Pavey et al. study [2] in which GENEActiv was deployed, sedentary, walking, running and stationary activities were classified with 93% accuracy. Using the same device, Zhang et al. could achieve accuracies as high as 97% deploying support vector machines (SVM) and decision trees [11]. There, the recognized activities were sedentary, household activities, walking and running. In a recent study, Mannini et al. reported a recognition rate of 89% for a group of youths and adults using wrist-worn sensors [12]. They declared that using triaxial Wocket accelerometers and the SVM algorithm, sedentary and ambulatory activities were properly differentiated in both youths and adults. GENEActiv was also used in another experiment made by Garcia-Ceja et al., where shopping, showering, working with a computer, eating meal, commuting, sports activities and brushing teeth were successfully classified with a maximum rate of 77% accuracy. There, the Hidden Markov model (HMM) and conditional random field were deployed for classification [13].

Ellis et al. successfully classified household activities, walking, jogging and stairs using a wrist-worn ActiGraph GT3X+ accelerometer complemented by HR measured by Polar RS400 with 88% accuracy utilizing the RF classifier [8]. The same author presented another wrist-based activity recognition framework in which they used the same device, but a multilevel classification approach using RF and HMM. They reached an 85% accuracy in classifying sitting, standing, walking/running and riding in a vehicle [9]. ActiGraph GT3X+ was also exploited in the study done by Trost et al. in which an average classification accuracy of 88% was achieved for activities including sitting, standing, walking, playing basketball, lying down, running and dancing. The regularized logistic regression model was used as the classification algorithm in that work [14].

Machine learning techniques have been widely used for activity monitoring [15]. Depending on the nature of the task, supervised or unsupervised algorithms can be employed. Supervised learning has been selected in the majority of the studies mostly because of the heterogeneity of human movement patterns. Supervised classification algorithms are hence suitable for activity recognition given that enough training data are available. Nowadays, collecting data is not an issue since numerous wearable IMUs are on the market with lower than ever prices.

In this work, we are aiming to show how accurately a single PPG-ACC wristband can detect different home-specific activities and in particular activities with varying intensities, but similar wrist motion patterns. There have not been many studies in which fusion of wrist-based HR and ACC were studied for detecting activities such as stationary cycling with varying intensity levels. Moreover, we present a framework comprised of the best pre-processing and parameter choices for an RF classifier. The framework can then be replicated by other researchers and practitioners in the future.

## 3. Materials and Methods

### 3.1. Data Collection

A PulseOn CLOUD PPG-based heart rate monitoring wristband (PulseOn, Espoo, Finland) with an embedded triaxial accelerometer (range ±8*g*) was worn on the dominant wrist of the participants. The device collects both HR and ACC measurements with a 25-Hz sampling frequency. The wristband connects with mobile phones via Bluetooth connection for both real-time visualization of the HR and ACC values and data storage. The data can then be exported from the connected mobile phone in the form of relational databases.

Twenty-five healthy male adults (age = 28.1 ± 3.8 (years), body mass index (BMI) = 24.8 ± 3.4 (kg/m^2^)) participated in the study from which five were excluded from final analysis. Most of the recruited subjects were students at Tampere University of Technology, Finland. The excluded subjects had problems in either data storage (*N* = 3) or lack of adherence to the pre-trial instructions (*N* = 2). Inclusion criteria were having at least eighteen years of age and being ambulatory. Written informed consent was collected from all subjects before participation. Ethical clearance was obtained from The Academic Ethics Committee of the Tampere Region (Statement 35/2016).

The study protocol consisted of a single testing session in a furnished home-like apartment in which a set of typical home-specific activities were performed. The activities are described in Table 1.

### 3.2. Data Pre-Processing and Feature Extraction

After completion of the laboratory protocol, class labels were added to each subject’s data according to the time points that were recorded by the laboratory instructor during the tests. The time points at which each activity started and ended were written down by the laboratory instructor during the tests. Labels were then assigned to every sample of the data whose timestamp fell between the mentioned beginning and end time points. To correct the potentially inaccurate labels at the times of transitions from one task to another, the laboratory instructor visually inspected the graphs of ACC and HR signals and interactively adjusted the times at which the transitions occurred.

Although window length for ACC signals has been widely tested in the literature, there was not much evidence about the window length effect when features of HR and ACC signals are combined. Hence, the pre-processed data were segmented into 1-, 3-, 5-, 7-, 9-, 11-, 13-, 15-, 17-, 19- and 21-s time windows with 90% overlap. To recognize the activities on a real-time basis, the HAR system must deliver the labels of activities frequently enough. This is the reason for having 90% overlap between every two adjacent windows in this study. Apart from that, Janidarmian et al. [15] showed that their HAR system achieved the highest recognition accuracies when 90% overlap was deployed.

From ACC signals, both time and frequency domain features were extracted. In the time domain, features were the mean and standard deviation of the absolute value of each axis. Absolute values were used since the subjects were wearing the device on either the left or right wrists depending on which one was their dominant hand. From the three ACC axes, signal magnitude area (SMA), mean, standard deviation, median, mean absolute deviation and the median absolute deviation of signal magnitude vector (SMV), as well as correlations between each pair of the three axes were calculated [6]. In the frequency domain, the relative energy of each axis in different frequency bands ((0–1],(1–2],(2–3],(3–4],(4–5],(5–6],(6–7],(7–8],(8–9],(9–10],(10–12.5) Hz) was computed for each window using short-time Fourier transform (STFT). In addition, the dominant frequency and its magnitude in the whole frequency range (0–12.5 Hz) of each axis in each window were computed.

From the HR signal, time domain features including mean, standard deviation, mean difference of consecutive windows, mean difference of every second window, mean absolute deviation and median absolute deviation were extracted [6].

To remove the redundancies and potentially correlated features in the feature set, pair-wise Pearson correlation coefficients of all the features were computed, and from the pairs whose coefficients were greater than or equal to 0.95, one feature was excluded from the whole feature set. At the end, 4 pairs of correlated features were recognized, and from each pair, one of the features was excluded from the feature set. Altogether, 62 features were present in the final feature set.

All the signal segmentation and feature extraction work was done using R Version 3.1.2 [17].

### 3.3. Classification Algorithm: Random Forest

Random forest is a statistical learning algorithm employing a large ensemble of decision trees [7] used for both regression and classification tasks. Remarkable classification performance along with relatively simple training and tuning are the strengths of the RF algorithm [18].

In the case of classification, the RF algorithm employs a set of classification trees, while each tree is built on a bootstrapped sample of the original data [7]. The classification trees are built based on recursive binary splits, as for each split, a randomly-chosen subset of input variables is used to find the best binary split [19]. In each tree, the best splits are determined using the Gini index values [19].

The RF algorithm can be used for feature selection, as well [7]. This is done via measuring the mean decrease of accuracy when a particular feature is removed from the set of features in the trees. If the accuracy deterioration after feature exclusion is negligible, the feature is less important and vice versa. The RF algorithm assigns the importance score to each feature using the aforementioned concept. The importance scores of the features in the RF classifier [7,20] can therefore be evaluated and used as a feature selection criterion.

The function "RandomForestClassifier" [21] in the Python Scikit Learn package [22] was used for constructing the RF classifier in this study.

### 3.4. Analysis Framework

We designed four experiments in which the effect of window length, forest size, HR features and the utility of each single feature in classification performance were comprehensively analyzed. Under the Results Section, in Experiment 1, the effect of window length on classification performance is presented. Next, in Experiment 2, the relation between the number of trees in the forest and the classification performance of the RF classifier is shown, considering that it is computationally favorable to deploy as few trees as possible. In Experiment 3, the utility of HR features in the performance of the classifier is demonstrated. Finally, in Experiment 4, the importance of each feature and its effect on the classification performance is investigated.

The hyper-parameters of the RF classifier [22] were all constant throughout the analysis except the ones with which we were experimenting. The values of hyper-parameters were as follows. Gini index values were used for selecting the best split. The maximum number of features considered for identifying the best split was set to the square root of the total number of features in the feature set. The maximum allowed depth of the trees in the forest was set to either pure leaves or all leaves having less than 2 samples, which was the minimum number of samples required to split an internal node. The minimum number of samples required to split an internal node was set to 2. The minimum number of samples at a leaf node was set to 1. Bootstrap samples were used for building the trees. Tree-specific balanced class weights were deployed to alleviate the effect of the imbalance of the class sizes when a bootstrap sample was selected for each tree.

In all stages of the analysis, the weighted mean value of LOOCVs was used as the performance metric. The weighted mean was exploited to account for the imbalance of the class sizes. The experiments were all done by the Anaconda distribution of Python 3.6.0. The following section includes the four above-mentioned experiments.

## 4. Results

### 4.1. Experiment 1: Effect of Window Length

**Figure 1:**
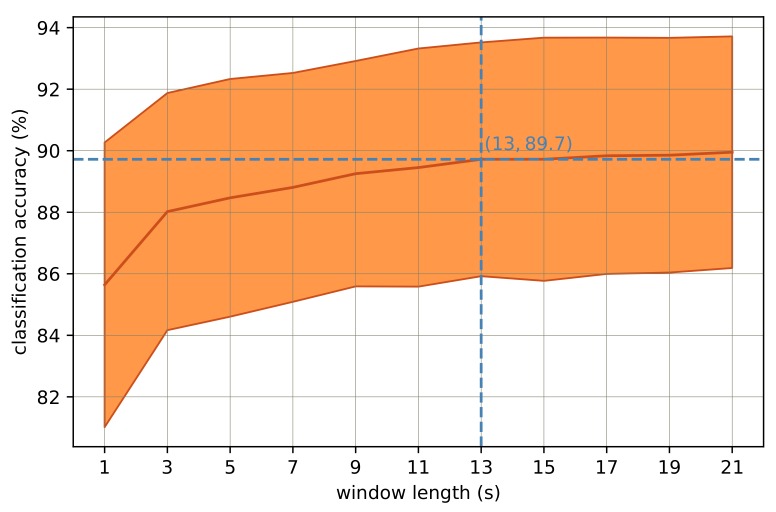
Classification accuracy versus window length in seconds. The shaded areas highlight ± one standard deviation from the mean value.

The effect of window length on recognition accuracies was explored deploying an RF classifier with a sufficiently big forest size, 128 trees, as stated in [23]. The classifier was separately trained and tested for feature sets with 1-, 3-, 5-, 7-, …, 21-s window length values. For each window length, the results of LOOCV are sketched in Figure 1. As can be seen, the classification accuracies increased from the 1-s window until the 13-s window, when it plateaus afterwards. The best window length is therefore 13 s, and the results of the next experiments are all based on the feature set with a 13-s window length.

### 4.2. Experiment 2: Effect of Forest Size

**Figure 2:**
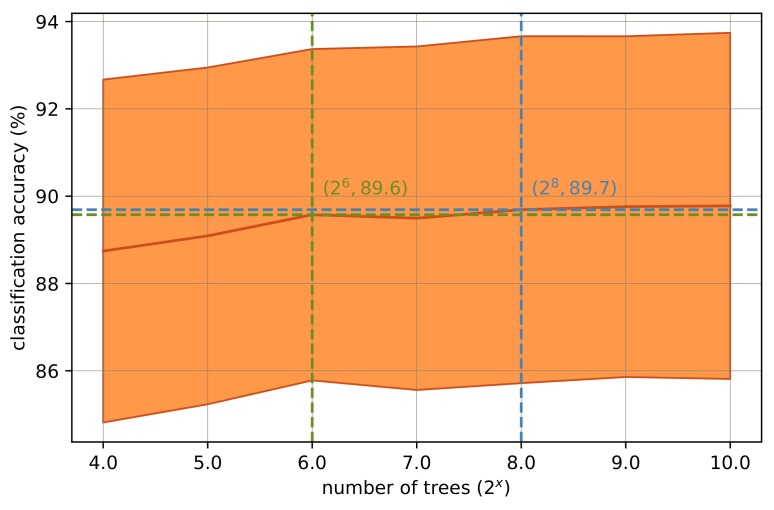
Classification accuracy versus the binary logarithm of the number of trees. The shaded areas highlight ± one standard deviation from the mean value.

The computational complexity and the classification accuracy of the RF classifier are both affected by the forest size. Although a forest of a size of 128 was shown to be sufficient for a number of medical/biomedical classification problems [23], we tested the forest size effect on classification performance while data with 13-s signal segments were used. Forest sizes of 16, 32, 64, …, 1024 were separately deployed, and correspondingly, an RF classifier was trained and tested. The results can be seen in Figure 2. Here it was shown that even with a forest of a size of 64, the classifier achieved high enough accuracy. Although the maximum recognition accuracy was achieved with 256 trees, the difference between recognition rates corresponding to 64 trees and 256 trees is in the order of 0.1%. Therefore, in all the next experiments, a forest size equal to 64 has been used.

### 4.3. Experiment 3: Effect of HR Features

**Figure 3:**
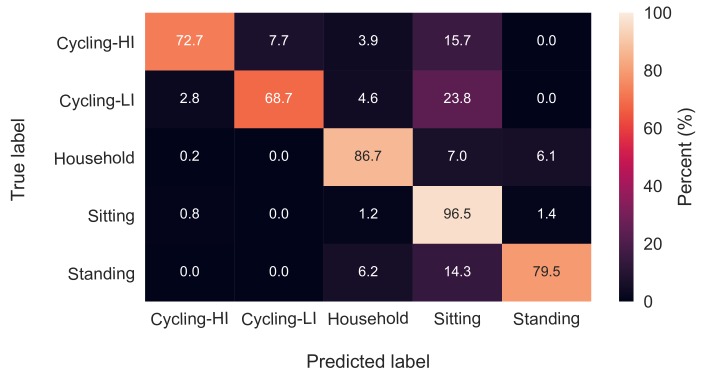
Confusion matrix of classification with both HR and ACC features.

**Figure 4:**
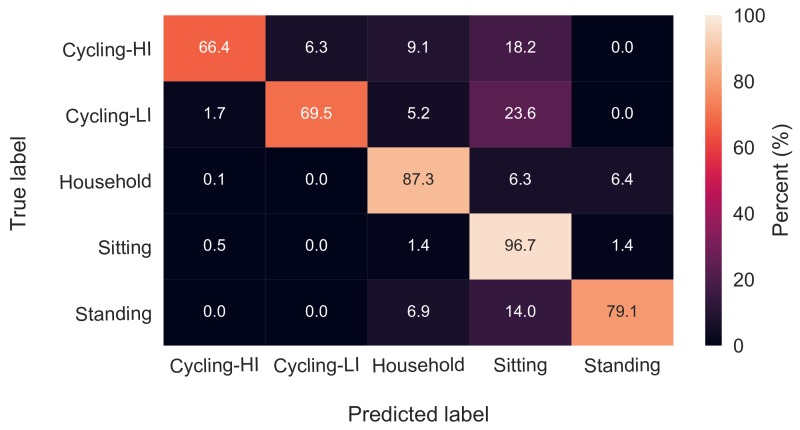
Confusion matrix of classification with only ACC features.

The effect of HR features on classification accuracies was examined using an RF classifier with 64 trees and the feature set with a 13-s window length. The mentioned classifier was once trained and tested while including all the HR and ACC features and another time with only ACC features. The overall accuracy values were 89.6 ± 3.9% and 89.3 ± 3.9% for with-HR and without-HR feature sets, respectively. The classification results are depicted in the two confusion matrices in Figure 3 and Figure 4. There were no significant changes in the classification accuracies of standing, sitting, household activities and low-intensity cycling categories, while the high-intensity cycling was classified with 7% less accuracy when HR features were excluded.

According to the confusion matrices in Figure 3 and Figure 4, sitting, household activities and standing were the categories with the highest recognition rates in both with-HR and without-HR feature sets. The erroneous classifications in both with-HR and without-HR feature sets were mostly due to misclassifying the three categories, standing, low-intensity cycling and high-intensity cycling as the sitting category with 14%, 24% and 20% confusion, respectively.

### 4.4. Experiment 4: Optimum Number of Features

**Figure 5:**
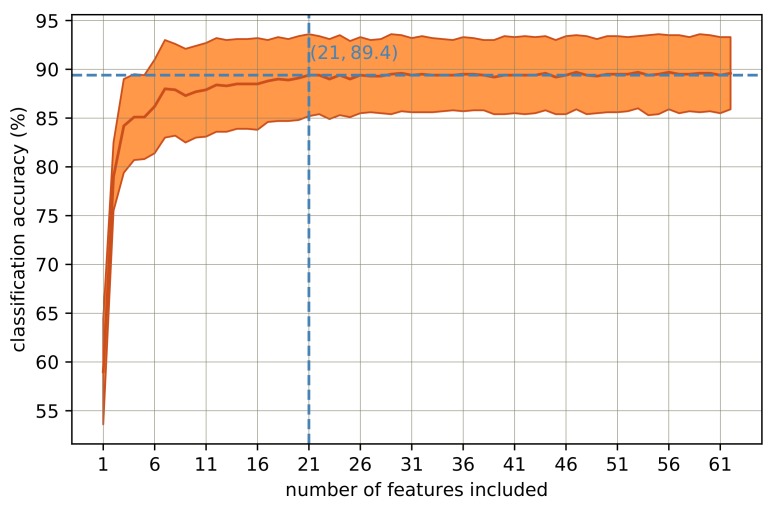
Classification accuracy of the RF classifier every time one feature was added to the feature set. The shaded areas show ± one standard deviation from the mean value.

The importance score of the RF classifier for each feature, as described in [7], can be used to determine the optimum number of features required for a proper classification. An RF classifier with 10,000 trees was trained, and feature importance scores were extracted. Deploying 10,000 trees is required to reliably extract feature importance scores [7]. The features were then sorted based on their importance scores descendingly. Next, an RF classifier with 64 trees, as proven in Experiment 2, is trained and tested iteratively by growing the feature set with one feature at a time from the above-mentioned sorted list of features. That is, in the very first iteration, the feature set included just one feature, which has the highest importance score. In the second iteration, the two features with the highest importance scores were included in the feature set. Similarly, the feature set was grown repeatedly with one feature added at a time from the above-mentioned list of sorted features. Figure 5 shows how the overall LOOCV accuracy changed by growing the feature set from Feature Number 1 all the way until Feature Number 62.

**Figure 6:**
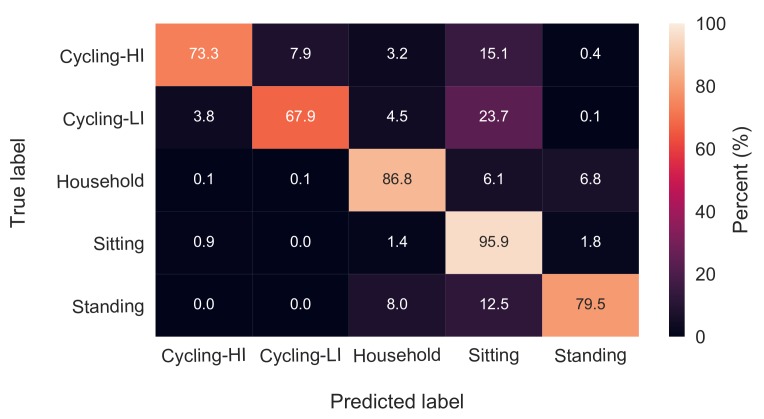
Confusion matrix of classification with the shrunken set of features that included both HR and ACC features.

As can be seen from Figure 5, the classification accuracy rose to almost its peak value of 89.4% when the feature set was expanded until Feature Number 21. In other words, exploiting the 21 most important features, the classifier converged to its peak performance. The performance of the classification algorithm was then retested using only the 21 features. This has resulted in 89.4 ± 4.2% accuracy and the confusion matrix depicted in Figure 6. The 21 features and their corresponding importance scores are described in Table 2. As explained before, to see the effect of each feature, the feature set was grown repeatedly by one feature at a time. Accordingly, in Table 2, the accuracy values of the RF classifier corresponding to the grown feature set from Feature Number 1 all the way until Feature Number 21 are depicted.

## 5. Discussions

In this study, a set of human activities recorded by a single PPG-ACC wrist-worn sensor was classified deploying an RF classifier. The aim of the study was to comprehensively examine (1) how accurately the human activities can be classified; (2) how the signal segmentation affects the overall classification results; (3) the optimum number of RF trees required for HAR purposes; (4) the effect of HR features on classification results and in particular recognition rates of varying intensities of a same activity type; and (5) the optimal number of features by which the peak performance can be achieved.

The recognized activities were typical home-specific daily tasks including: (1) sitting (sitting still, sitting and drinking and sitting and doing math without pen and paper); (2) standing still; (3) household activities (two types of dish washing and two types of table cleaning); (4) low-intensity stationary cycling at 60 rpm; and (5) and high-intensity stationary cycling at 90 rpm [6]. All the activities were performed in a home-like apartment, which provided more realistic results over laboratory environments.

The effect of signal segmentation (or window length) on activity classification was addressed by assessing a range of different window length values. The window length values were 1, 3, 5, …, 21 s. According to Figure 1, the maximum classification accuracy could be obtained by the 13-s signal segments. In this study, the adjacent signal segments had 90% overlap. Such a high overlap value was selected because in a real-time activity recognition system, the activity labels must be delivered as frequently as possible. Although deploying overlapping windows results in more training samples, in practice, not much improvement is made in the overall performance due to the correlation of the data [24]. Deploying 90% overlapped segments for short window lengths (e.g., less than 5 s), is not required however; because the time duration between every two labels would not be much and the labels are delivered frequent enough. However, there is no harm in deploying overlapping windows from the classification point of view, although it is computationally more expensive. Thus, we tended to keep the pre-processing approach the same for all signal segment lengths despite the burden of computational complexity.

The signal segmentation effect was extensively studied in many papers. Mannini et al. reported an 85% LOOCV recognition accuracy using a non-overlapping 12.8-s window length for wrist-based ACC signals. In the same work, non-overlapping 4-s windows were also tested and showed an 84% LOOCV classification accuracy, which is only 0.05% less than the case with 12.8-s windows [24]. Mannini et al. proposed 4-s windows for real-time activity recognition purposes as the 4-s latency is tolerable for real-time applications. Zhang et al. deployed non-overlapping 12.8-s windows and obtained 96% and 97% overall 10-fold cross-validation accuracies for left and right wrists [11]. The best window length obtained in the aforementioned two works is considerably similar to what has been presented in this work, stating that a 13-s window length could provide the best classification results. Deploying longer time windows may however lead to better results when HR features are also taken into account, mainly because of the latency of the cardiovascular system in stabilizing HR after transiting from one task to another. Lengthening the signal segments beyond 13 s may, however, lead to overlooking the short-lasting activities and therefore less accuracy. Concerning the classification accuracies, Mannini et al. tended to impose as little instruction as possible on the study participants [24]. They were able to achieve moderately high accuracy levels, but still not as good as what has been presented by our work in this paper. Zhang et al. got significantly higher accuracy rates; however, they did not tend to discriminate varying levels of the same activity type as they stacked all the same activity types into one broad category [11]. On the other hand, 10-fold cross-validation was deployed for the validation of their models, which is in principle subject dependent and normally produces higher classification rates compared to LOOCV.

The forest size was assessed in this study comprehensively after the determination of the best window length. Different number of trees from the set of 16, 32, 64, …, 1024 were tested, and the results are shown in Figure 2. The best results were obtained using 256 trees, whilst almost as good results could also be acquired using 64 trees. A fewer number of trees is preferred as it ends up with less computational complexity [23].

The effect of HR features was revealed when the RF classifier was trained once with and once without HR features. There was no effect on the recognition rates of household activities, sitting, standing and low-intensity cycling, while the high-intensity cycling was classified with almost 7% less accuracy when HR features were excluded. Ellis et al. stated that the effect of HR in activity recognition is not significant [8], although their study did not include tasks having different intensities, but similar hand motions, such as cycling with different intensities in this study. In the studies of Rosenberger et al. [25] and Mannini et al. [24], the most difficult task to classify was cycling mostly due to the stationary position of the hands on the bike handle bar, which resembles sedentary activities. By introducing HR features to the classification algorithm in our study, not only the cycling tasks were reasonably identified, but also their intensities were recognized with almost 70% accuracy as shown in Figure 3. According to Table 2, the mean value of HR was the third most important feature among all the extracted features. However, activity recognition may not be done solely based on HR due to the latency of the cardiovascular system in responding/recovering to/from changes in the activities. For instance, HR recovers slowly after some minutes of moderate exercise [26]. Such a latency in HR recovery is probably the reason for it being not the most important feature in Table 2.

In order to manage feature reduction, the feature importance score of the RF classifier was used. To avoid biased scores [7,20], first highly correlated pairs of features were identified, and from each pair, one feature was excluded from the feature set. Next, a huge forest with 10,000 trees was trained on the data [7]. It should be noted that there was no categorical variable in the feature set, which would have biased the scores. All the features were transformed to zero-mean and unit-variance prior to training the classifier. Using the importance scores, a shrunken feature set of only 21 features was obtained. The confusion matrix of classification with the selected 21 features is depicted in Figure 6. Comparing the confusion matrices in Figure 3 and Figure 6, there is no considerable accuracy deterioration owing to feature reduction. The 21 features presented in Table 2 can be therefore replicated for similar purposes.

### Weaknesses, Strengths and Future Work

One of the weaknesses of this study is having a homogeneous and relatively small sample of young subjects. Moreover, the study protocol is lacking activities such as walking and running mostly due to the fact that the primary aim of the study was testing a set of home-specific activities. Classification rates could benefit from second-by-second labels instead of one label for the whole duration of each activity. Activities like dish-washing and table cleaning are composed of short segments of standing still, as well. Having second-by-second labels would allow reducing the misclassifications of these kinds of activities.

The strengths of the study include data collection in a real home environment with very little administrative restrictions on the users. This has led to closer to reality data and more generalizable results. The reproducibility of the study is quite high as the activity recognition was done exploiting only one off-the-shelf wrist-worn device. No data cleaning or filtering of any kind was done on the data before signal segmentation. Such an approach requires less computation and therefore lower latency for real-time applications. In plenty of similar studies, the data at the beginning and the end of the tasks were discarded, while in this study, we tended to keep all of the data, which makes the classification more challenging. The results shown in this study were all acquired with leave-one-subject-out cross-validation, which is an unbiased validation approach that provides subject independent validations.

The future work could include implementing an adaptive window length selection approach that shortens the signal segments at the time of transitions from one task to another. This will probably lead to higher classification rates. In addition, the classification accuracy could benefit from employing different window lengths for different parameters, as the ACC and HR features have quite different dynamics. Furthermore, in the future, it would be worth trying to implement and test the proposed framework on embedded systems and observing the performance when it is used for real-time activity recognition.

## 6. Conclusions

An activity recognition framework deploying a single wrist-worn optical HR monitoring and triaxial accelerometer was presented in this paper. The random forest classifier was used to recognize the patterns of each activity. Different parameters and hyper-parameters affecting the classification performance were comprehensively examined, and the best options were reported. Wrist-worn HR monitoring sensors were shown to be suitable for activity monitoring purposes and specifically advantageous in recognizing identical activities with varying intensities. Activities with varying levels of intensities are sometimes challenging to discriminate mainly due to the similarity of the wrist movements. In this work, there was stationary cycling with two levels of difficulty. Complimenting the ACC data with HR has clearly benefited the discrimination of stationary cycling with two intensity levels. The presented framework could be replicated for similar use cases.

## Figures and Tables

**Table 1:** The set of activities included in the study protocol. Those subjects who were physically active (activity class ≥5, range 1–10) were instructed to cycle at a 100-Watt power level, while those who were less physically active (activity class <5, range 1–10) were instructed to cycle at 50-Watt power level [16].

Activity Type	Activity Sub-Type	Duration (min)
Sitting	sitting still	2 × 5
	sitting and drinking	5
	sitting and doing math	5
Standing	standing still	2 × 5
Household activities	dish washing	2 × 3
	table cleaning	2 × 3
Stationary cycling	high intensity	3
Stationary cycling	low intensity	3

**Table 2:** The 21 most important features along with their corresponding importance scores (IS), as well as the average percentage of classification accuracies (Avg. Accuracy) expressed as the mean ± standard deviation. By moving from top rows to the bottom, the feature set was grown by one feature at a time.

	Feature	Rounded IS	Avg. Accuracy (%)
1	Mean of absolute value of ***Y*** axis	0.09	59.0±5.4
2	Median absolute deviation of SMV	0.08	79.0±3.5
3	Mean of HR	0.07	84.2±4.8
4	Mean absolute deviation of SMV	0.05	85.1±4.4
5	Mean of absolute value of ***X*** axis	0.04	85.1±4.3
6	Total power of ***Y*** axis	0.04	86.2±4.8
7	Dominant frequency in ***Y*** axis	0.04	88.0±5.0
8	Standard deviation of absolute value of ***Y*** axis	0.04	87.9±4.7
9	Mean of absolute value of ***Z*** axis	0.03	87.3±4.8
10	Total power of ***Z*** axis	0.03	87.7±4.7
11	Mean of SMV	0.03	87.9±4.8
12	Dominant frequency in ***X*** axis	0.03	88.4±4.8
13	Spectral power of dominant frequency in ***Y*** axis	0.03	88.3±4.7
14	Standard deviation of absolute value of ***X*** axis	0.02	88.5±4.6
15	Total power of *X* axis	0.02	88.5±4.6
16	Standard deviation of absolute value of ***Z*** axis	0.02	88.5±4.6
17	Spectral power of *Y* axis in band (0–1] Hz	0.02	88.8±4.2
18	Spectral power of *X* axis in band (0–1] Hz	0.02	89.0±4.3
19	Spectral power corresponding to dominant frequency in ***X*** axis	0.02	88.9±4.2
20	Spectral power corresponding to dominant frequency in ***Z*** axis	0.01	89.1±4.3
21	Cross-correlation coefficient of ***X*** and ***Z*** axes	0.01	89.4±4.2
